# Involvement of the *Azotobacter vinelandii* Rhodanese-Like Protein RhdA in the Glutathione Regeneration Pathway

**DOI:** 10.1371/journal.pone.0045193

**Published:** 2012-09-25

**Authors:** William Remelli, Nicoletta Guerrieri, Jennifer Klodmann, Jutta Papenbrock, Silvia Pagani, Fabio Forlani

**Affiliations:** 1 Dipartimento di Scienze per gli Alimenti, la Nutrizione e l'Ambiente, Università degli Studi di Milano, Milano, Italy; 2 Institut für Pflanzengenetik, Leibniz Universität Hannover, Hannover, Germany; 3 Institut für Botanik, Leibniz Universität Hannover, Hannover, Germany; National Institute for Medical Research, Medical Research Council, London, United Kingdom

## Abstract

The phenotypic features of the *Azotobacter vinelandii* RhdA mutant MV474 (in which the *rhdA* gene was deleted) indicated that defects in antioxidant systems in this organism were related to the expression of the tandem-domain rhodanese RhdA. In this work, further insights on the effects of the oxidative imbalance generated by the absence of RhdA (e.g. increased levels of lipid hydroperoxides) are provided. Starting from the evidence that glutathione was depleted in MV474, and using both *in silico* and *in vitro* approaches, here we studied the interaction of wild-type RhdA and Cys^230^Ala site-directed RhdA mutant with glutathione species. We found that RhdA was able to bind *in vitro* reduced glutathione (GSH) and that RhdA-Cys^230^ residue was mandatory for the complex formation. RhdA catalyzed glutathione-disulfide formation in the presence of a system generating the glutathione thiyl radical (GS^•^, an oxidized form of GSH), thereby facilitating GSH regeneration. This reaction was negligible when the Cys^230^Ala RhdA mutant was used. The efficiency of RhdA as catalyst in GS^•^-scavenging activity is discussed on the basis of the measured parameters of both interaction with glutathione species and kinetic studies.

## Introduction

Sulfurtransferases catalyze *in vitro* the transfer of a sulfur atom from a suitable sulfur donor (thiosulfate for rhodaneses, and 3-mercaptopyruvate for 3-mercaptopyruvate sulfurtransferases) to cyanide, with concomitant formation of thiocyanate [1]. The first characterized sulfurtransferase was bovine liver rhodanese which has been the subject of numerous investigations [1]–[4]. By genome sequence analysis, more than 9000 ORFs, displaying domains similar to the bovine liver rhodanese domains, have been gathered into the homology superfamily of the rhodanese-domain proteins (Pfam acc. n.: PF00581; http://pfam.sanger.ac.uk/). Analysis of sequence databases revealed that rhodanese domains can be fused to domains of distinct function, or unknown function, an evidence supporting their ability to interact with other proteins. The reduced form of thioredoxin was shown to be a good sulfur acceptor substrate in the *in vitro* sulfur transfer catalyzed by the bovine liver rhodanese [3], the mouse mammary gland 3-mercaptopyruvate sulfurtransferase [5], the leishmanial 3-mercaptopyruvate sulfurtransferase [6], the *Escherichia coli* GlpE [7], and the *Trichomonas vaginalis* 3-mercaptopyruvate sulfurtransferase [8]. In *Arabidopsis thaliana*, preliminary work was carried out by bimolecular fluorescence complementation indicating specific interactions among sulfurtransferases and thioredoxins of the same cell compartment [9]. These lines of evidence support the notion that cyanide detoxification [1] is not the only physiological role of the rhodanese-like proteins. The difficulty to define different roles of these proteins present in all domains of life is predominantly due to their presence as paralogs in the majority of organisms. The wide variability in the amino acids of their active-site loops [10] suggests that substrate recognition and biological interactions are driven by specific active-site structures.

**Figure 1:**
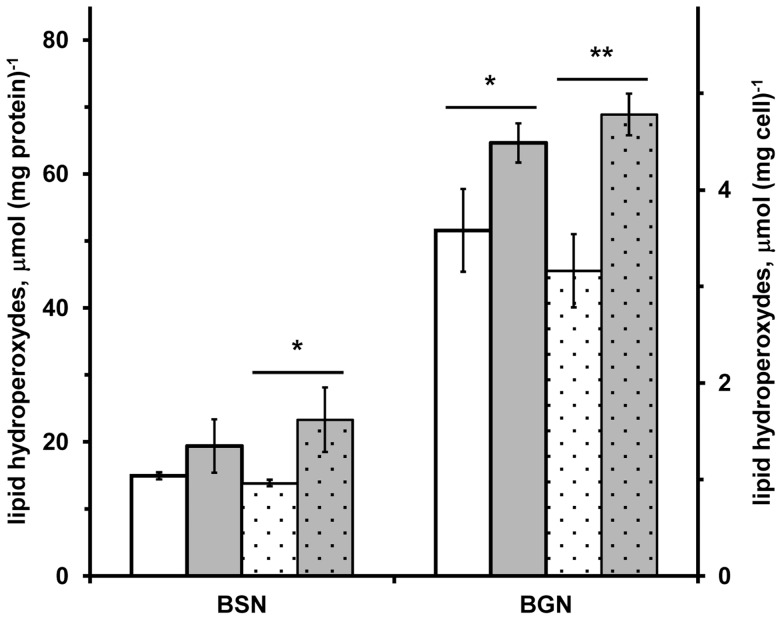
Lipid hydroperoxide concentration in *A. vinelandii* cells. Lipids hydroperoxides were detected, after incubation with FOX 2 reagent, in wild-type (UW136; white bars) and in *rhdA* null mutant (MV474; grey bars) *A. vinelandii* strains grown in Burk's medium containing sucrose (BSN) or gluconate (BGN) as carbon source. Data are normalized relative to the cell mass (dotted bars) and to the protein amount (not-dotted bars) detected in 10 mM Tris-HCl 0.1 M NaCl (pH 8) extracts of cell-samples from the same growths, and are the mean of three independent replicates ± standard deviation. Differences significant for *P*<0.05 (*) or *P*<0.01 (**) are indicated (Student's *t* test).

In the present work, the effects of the oxidative imbalance generated by the absence of RhdA in *A. vinelandii* were further defined, and, starting from the evidence that in the MV474 RhdA-null mutant strain the level of glutathione disulfide (GSSG) was higher with respect to that measured in the wild-type strain [15], we investigated a possible link between RhdA and homeostasis of reduced glutathione (GSH). We found that RhdA was able to bind *in vitro* GSH and to a lesser extent GSSG. RhdA catalyzed GSSG formation in the presence of a system generating the glutathione thiyl radical (GS^•^, an oxidized form of GSH), thereby facilitating the conversion of GS^•^ to GSSG, a GSH-generable form, and the RhdA-Cys^230^ residue was mandatory for the GS^•^-scavenging activity of RhdA. Taking into account that GS^•^, generated by reaction of GSH with cell radicals (e.g. ^•^OH), can have high pro-oxidant activity and can be modified in other radical forms by overoxidation or by intramolecular hydrogen transfer [24], [25], the relationship between the phenotypic features of the RhdA mutant and the *in vitro* results of RhdA GS^•^ scavenging activity is discussed.

**Figure 2:**
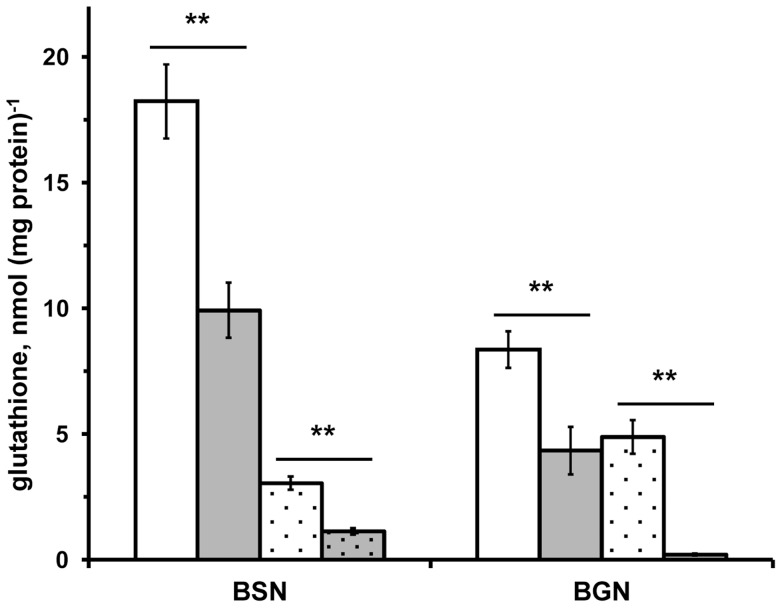
Glutathione in *A. vinelandii*. (**A**) Glutathione was detected in wild-type (UW136; white bars) and in RhdA-null mutant (MV474; grey bars) *A. vinelandii* strains grown in Burk's medium containing sucrose (BSN) or gluconate (BGN) as carbon source in the absence (not-dotted bars) and in the presence (dotted bars) of phenazine methosulfate (PMS). Measured glutathione includes both the total reduced-glutathione and the DTT-reducible fraction of total free glutathione and is expressed as function of the protein amount detected in 10 mM Tris-HCl 0.1 M NaCl (pH 8) extracts of cell-samples from the same growths. All data are the mean of three independent replicates ± standard deviation. Differences significant for *P*<0.01 (**) are indicated (Student's *t* test).

**Figure 3:**
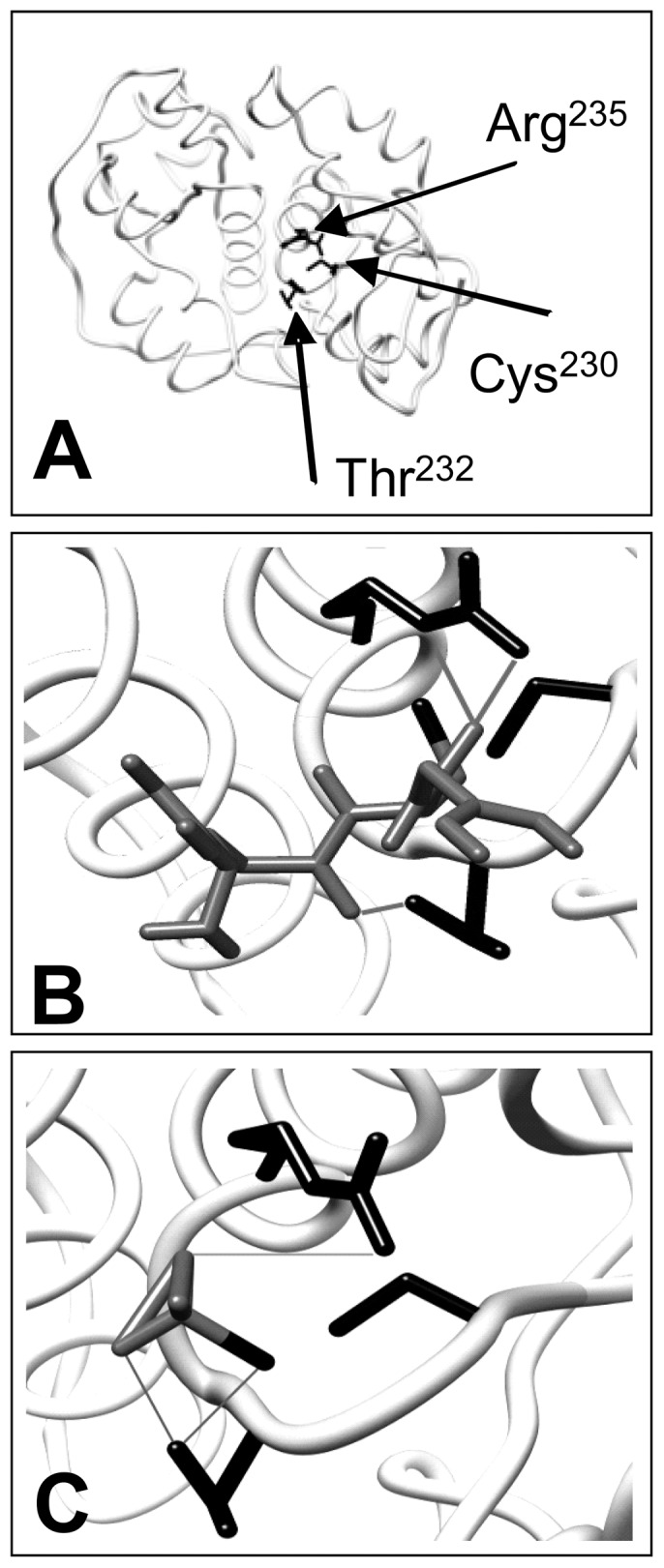
*In silico* docking of glutathione and thiosulfate to RhdA. Overall RhdA structure (**A**) and its active site region interacting with either reduced glutathione (**B**) and thiosulfate (**C**). The amino acidic residues (Arg^235^, Thr^232^ and Cys^230^) involved in the interactions are highlighted in black. Ligand backbones are displayed as dark grey sticks. *In silico* computed hydrogen bonds between ligands and amino acidic residues are displayed as light grey solid lines. Images of molecular docking has been generated using UCSF chimera software (http://www.cgl.ucsf.edu/chimera/).

**Figure 4:**
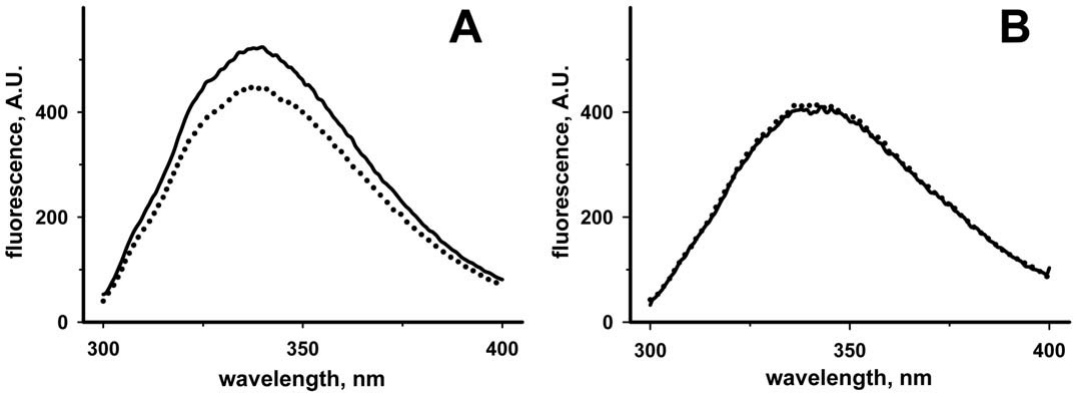
Intrinsic fluorescence changes of RhdA and RhdA-Cys^230^Ala in the presence of GSH. Tryptophan fluorescence emission spectra (λ_ex_ = 280 nm; dotted lines) of RhdA (**A**) and RhdA-Cys^230^Ala (**B**) in the presence of 50 µM GSH. Solid lines report spectra before the addition of GSH. Both RhdA and RhdA-Cys^230^Ala were 2 µM in 50 mM Tris-HCl, 100 mM NaCl (pH 7.4). Spectra were corrected for dilution.

## Methods

### Bacterial strains and growth conditions

The *A. vinelandii* strains used in this study were UW136 and a derivative of UW136 (MV474), in which disruption of the *rhdA* gene was achieved by the insertion of a KIXX (kanamycin resistance) cassette, following deletion of 584 bp as described previously [11]. Cells were grown for 24 h at 30°C under aerobic conditions in liquid Burk's medium [26] supplemented with 1% (w/v) sucrose (BSN) or 0.2% (w/v) gluconate (BGN) in the presence of 15 mM ammonium acetate. When the absorbance at 600 nm reached 2.0, the cells were centrifuged at 3800× *g* for 30 min and stored at −80°C. For oxidative-stress sensitivity assays, cells were grown in the above conditions up to the absorbance value (at 600 nm) of 0.8, then the cultures of either UW136 or MV474 strains were divided into two equal samples, one of which was treated with 15 μM phenazine methosulfate (PMS).

For protein overexpression, the *Escherichia coli* strain, BL21[pRep4] harbouring overespression plasmid, was grown in Luria-Bertani medium (containing 100 µg/ml ampicillin and 25 µg/ml kanamycin) at 37°C and, when absorbance at 600 nm was 0.4, 1 mM isopropyl β-D-thiogalactoside was added. After further 4 h growth, cells were collected and used for protein purification.

### Protein preparations

His-tagged RhdA and RhdA-Cys^230^Ala were expressed in *E. coli* strains (BL21[pRep4]) harbouring pQER1 [27] and pQER1MP [13] respectively, and purified by Ni-NTA affinity chromatography [13]. Sulfane sulfur-deprived RhdA was prepared by cyanide treatment as previously described [28] and used for all experiments described in this work.

### Glutathione-thiyl radical preparation

Glutathione-thiyl radical (GS^•^) was prepared starting from reduced glutathione (GSH) using the horseradish peroxidase (HRP) generating system as described in Sipe et al. [29] with minor modifications. Reaction mixture, containing 90 nM HRP (type II, 188 U mg^−1^; Sigma), 0.5 mM phenolphthalein, 0.25 mM H_2_O_2_, and 5 mM GSH in 50 mM phosphate buffer (pH 7.4), was incubated for 2 min at room temperature and immediately used for enzymatic assays. The concentration of generated GS^•^ was assumed to be equal to the initial GSH concentration.

### Determination of lipid hydroperoxides

Lipid hydroperoxides (LHPOs) were quantified on total lipid extract according to a method based on the use of FOX 2 reagent [30]. One-hundred mg (fresh weight) of *A. vinelandii* cells were suspended in 1.9 ml of (1∶2 CHCl_3_/MetOH) by vortexing for 3 min. Then 0.63 ml of CHCl_3_ and 0.63 ml of H_2_O were added, and the sample was vortexed for 5 min. Phase separation was facilitated by centrifugation (5 min, 3800× *g*, 4°C). The organic phase was collected and washed twice with 2 volumes of H_2_O. For LHPO quantification, aliquot of organic phase (200 µl) was mixed with 1 ml of FOX 2 reagent, and incubated in the dark for 1 h at room temperature. FOX 2 reagent was freshly prepared before every assay. LHPO amount was determined spectrophotometrically (λ = 560 nm) using the molar absorption coefficient of linoleate hydroperoxide (ε = 6×10^4^ M^−1^ cm^−1^).

### Determination of intracellular glutathione

Measurement of the levels of glutathione was performed by a monobromobimane HPLC method [31] using 50 mg (fresh weight) cell samples. This method measures cumulatively both the dithiothreitol-(DTT-) reducible and the reduced forms of free glutathione.

### Enzymatic assay of glutathione-thiyl radical scavenging activity

GS^•^-scavenging activity assay was performed according to Starke et al. [32] with minor modifications. Reaction mixture containing 0.1 mM NADPH, 0.8 U baker yeast glutathione oxido-reductase (GOR; 230 U mg^−1^; Sigma), various amounts of RhdA (1–18 nM) in 50 mM sodium-phosphate buffer (pH 7.4) (final volume 900 µl) was incubated for 5 min at 25°C. Reaction was initiated by addition of 100 µl of GS^•^ premade mixture (see: “Glutathione-thiyl radical preparation”). Controls omitting RhdA were performed in parallel. To calculate GS^•^-scavenging activity, the initial rate of NADPH oxidation (stoichiometric to glutathione disulfide formation) was monitored at 340 nm (ε = 6220 M^−1^ cm^−1^) and was subtracted with the rate of the control. For calculation of apparent kinetic parameters, initial rates of reaction as a function of GS^•^ concentration were measured, were subtracted with the rate of the control, and values of *K*_m_ and *V*_max_ were calculated using non-linear regression curve (SigmaPlot, SPSS Inc., Chicago, IL, USA). For anaerobiosis studies oxygen was substituted with argon. One unit (U) of GS^•^-scavenging activity is defined as the amount of enzyme that produces 1 nmol GSSG per min at 25°C.

### Mass spectrometry analyses

Proteins were desalted using C4 ZipTips (Millipore) following the standard protocol given by the company. For MS analysis, the protein (1 µM) in ESI buffer (50% acetonitrile, 50% Millipore water, 0.1% formic acid) was injected into the mass spectrometer (micrOTOF-Q II, Bruker Daltonics) with a flow rate of 3 µl min^−1^ using a syringe pump (KD Scientific) and the ESI sprayer from Bruker Daltonics with 0.4 bar nebulizer gas and 4 l min^−1^ dry gas heated to 180°C. Each sample was measured for 5 min and an average spectrum was calculated using the DataAnalysis software (Bruker Daltonics). The protein mass was determined using the charge state ruler of DataAnalysis.

### Spectrofluorimetric measurements

Fluorescence measurements were carried out in a Perkin-Elmer LS-50 instrument, and data were analyzed as previously described [27]. RhdA intrinsic fluorescence spectra experiments (λ_ex_ = 280 nm) were carried out at 25°C in 50 mM Tris-HCl, 100 mM NaCl (pH 7.4) in the presence of 2 µM RhdA, and 50 µM glutathione (GSH, GS^•^ or GSSG). Where it is stated, 16 µM RhdA was treated with 8-fold excess of GSH for 20 min and unbound glutathione was removed by a gel-filtration step (Sephadex G-50; 1.5×10 cm column) before fluorescence measurements. For the evaluation of the dissociation constant (*K*_d_) of RhdA/glutathione bindings, changes of intrinsic fluorescence (λ_ex_ = 280 nm, λ_em_ = 342 nm) were monitored after sequential additions of 0.1 µM glutathione species or thiosulfate. Figures of intrinsic fluorescence changes were normalized for protein concentration and assay volume. The values of *K*_d_ were calculated by fitting intrinsic fluorescence changes to a non-linear regression curve (SigmaPlot). For thermal stability experiments, change of the RhdA intrinsic fluorescence was monitored during a Peltier-driven heating ramp of 0.5°C min^−1^ until 65°C.

### Other analytical procedures

Protein concentration was determined by the Bradford assay [33] using bovine serum albumin as standard.

Docking analysis were performed with Argus Lab software (Argus Lab 4.0.1, Planaria software LLC, Seattle, WA, U.S.A.; http://www.arguslab.com), using Lamarckian genetic algorithm scoring functions with 0.1 Å grid resolution, a 15 Å-edged box centred on the RhdA-Cys^230^ residue, and the flexible ligand mode (other parameters were kept with default values). Docking analyses were performed against RhdA structure (pdb ID: 1h4k) as template.

Theoretical calculation of p*K*_a_ value of the RhdA-Cys^230^ residue was carried out using the program PROPKA 2.0 [34] by using the crystal coordinates of the deposited RhdA structure (pdb ID: 1h4k).

## Results

### Effects of ROS accumulation in the *A. vinelandii* RhdA-null strain

The internal oxidative problem and the ROS accumulation in *A. vinelandii* RhdA-null strain (MV474) previously demonstrated [14], [15], could trigger modifications of cellular components. Considering that peroxidation of lipids is one of the important intermediary events in free radical-induced cellular damage [35], we compared the content of lipid hydroperoxides (LHPOs) in *A. vinelandii* wild-type and MV474 strains. As shown in **Figure** **1**, lipid hydroperoxides significantly increased in the absence of RhdA. The growths in gluconate as carbon source led to more marked effect than the growths in sucrose for which no significant difference is shown when data of LHPO amount are normalised on protein amount. It seems that the defensive mechanisms [15], [36] activated in the MV474 strain, are not adequate to counteract cellular damage in the absence of RhdA in *A. vinelandii*.

Further effect of the oxidative imbalance occurring in the absence of RhdA was the evidence that in the mutant strain MV474 the ratio GSSG/GSH was higher with respect to that measured in the wild-type strain [15]. To survey the effects of RhdA deficiency in modulating the level of glutathione in the cells, we measured glutathione in various growth conditions (**Figure** **2**). According to the applied method, glutathione measurements were cumulative of both the reduced glutathione (GSH) and the DTT-reducible fraction of free glutathione. In the presence of sucrose as carbon source, the level of glutathione was higher than in *A. vinelandii* grown in gluconate, but in both growth conditions the level of glutathione was depleted in the MV474 strain (*p*<0.01). To investigate whether the low level of glutathione found in the MV474 strain could stem from DTT-irreversible oxidation of glutathione due to the absence of RhdA in *A. vinelandii*, we used an artificial condition. *A. vinelandii* growths were carried out, in the absence and in the presence of the redox-cycling agent phenazine methosulfate (PMS). The addition of PMS in the growth media caused a decrease of glutathione level in both strains (**Figure** **2**, see dotted bars). To evaluate the effect of PMS, data can be referred as glutathione recovery that is the percent ratios of glutathione measured in PMS-induced stress to that measured in standard growths. Glutathione recovery in the wild-type strain was significantly higher than that in the RhdA-null mutant (16.9% *vs*. 11.4% in sucrose, p<0.05; 58.5% *vs*. 4.8% in gluconate, p<0.01), suggesting the involvement of RhdA in counteracting DTT-irreversible glutathione oxidation that likely occurred in PMS-growth conditions. Considering that GSH determination was carried out in the presence of the strong reductant thiol, DTT, the low level of GSH recovery in the absence of RhdA could stem from DTT-irreversible modifications of the cellular glutathione, thus implying that glutathione is modified in unrecoverable forms by overoxidation [24] or other molecular rearrangements [25]. A comparable trend of RhdA-promoted glutathione recovery was found in sucrose growths, although the effect of RhdA absence was less marked. These results agree with the previous evidence [14], [15] indicating that the carbon source gluconate was “a stressful growing condition” in which some physiological imbalance occurs, thus eliciting the RhdA effects.

Taken together, the above data further evidenced that the rhodanese-like protein RhdA can function as a putative antioxidant system in *A. vinelandii*, and suggest a relationship between RhdA and glutathione homeostasis.

### *In silico* and *in vitro* analysis of RhdA/glutathione interaction

The observation that both cysteine and glutamate concentrations were not depleted in the mutant strain, and that the absence of RhdA did not affect glutathione reductase activity (**Table S1**), prompted us to investigate the question whether RhdA could favour GSH regeneration *via* interaction with glutathione species.

Simulations of the docking process were carried out using the RhdA structure model obtained by crystallographic measurements [12], [16] as a template, and *in silico* prediction of the interaction of RhdA with GSH was compared with that of thiosulfate, the *in vitro* sulfane-sulfur donor for sulfurtransferase activity. Targeting the docking on the RhdA active site region (**Figure** **3A**), the position and orientation of both ligands into the RhdA active site indicated an involvement of the Cys^230^ thiol, being the estimated distance between the sulfur atoms 2.7 Å for GSH (**Figure** **3B**) and 2.5 Å for thiosulfate (**Figure** **3C**). Results of the docking suggest the involvement of Thr^232^ and Arg^235^ residue in the formation of 3 H-bonds that stabilize the complex with either GSH or thiosulfate (**Figure** **3B, 3C**). *In silico* docking of GSH and thiosulfate into the active site of RhdA gave rise to complexes with dissociation free energy values of 5.29 (GSH) and 6.01 (thiosulfate) kcal mol^−1^ (22 and 25 kJ mol^−1^, respectively). Attempts to dock GSSG into the RhdA active site region did not generate relevantly-scored complexes. Therefore, the active site of RhdA appears properly structured to form a complex with GSH, and prompted us to investigate this interaction *in vitro*.

**Figure 5:**
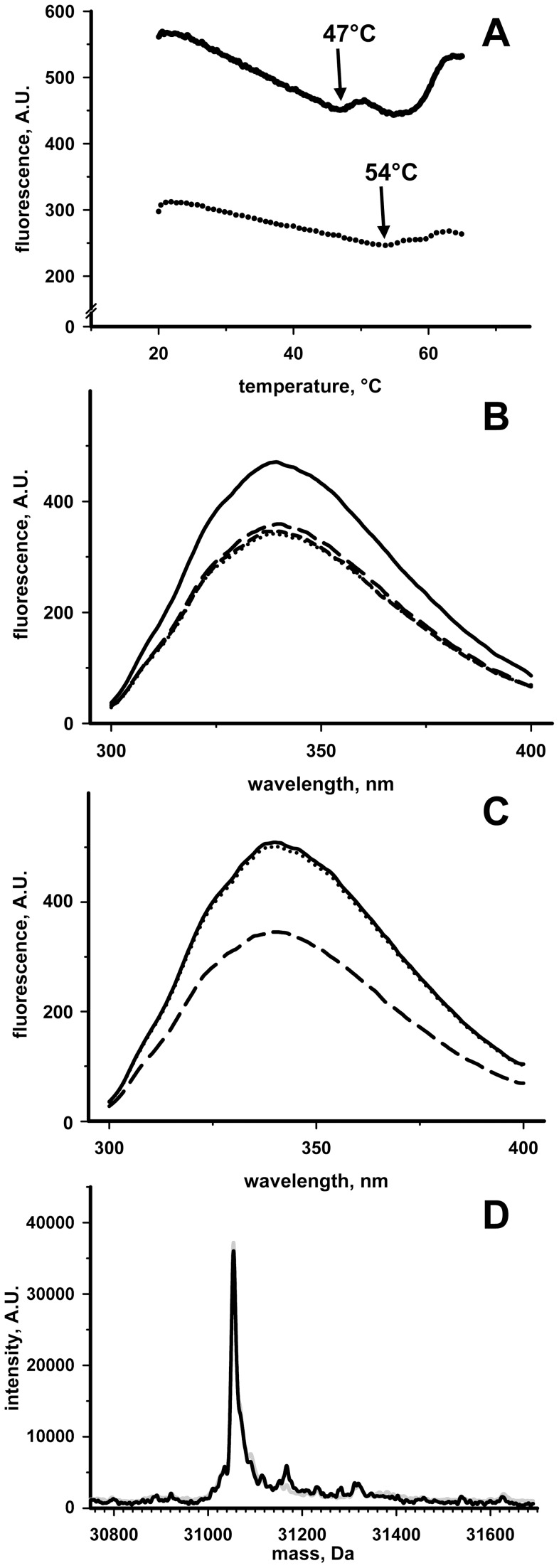
Analyses of the RhdA/GSH interaction. (**A**) Thermally-induced fluorescence changes of 2 µM RhdA (solid line) and RhdA/GSH complex (dotted line) in 50 mM Tris-HCl, 100 mM NaCl (pH 7.4). Tryptophan fluorescence was monitored as a function of the indicated temperature. Transition temperatures of the first conformational change are indicated. (**B**) Tryptophan fluorescence emission spectra (λ_ex_ = 280 nm) of 2 µM RhdA/GSH complex alone (dotted line) in 50 mM Tris-HCl, 100 mM NaCl (pH 7.4), in the presence of 0.8 mM Tris(2-carboxyethyl)phosphine (TCEP; long-dashed line), and in the presence of 0.2 mM DTT (short-dashed line). For RhdA/GSH complex preparation, RhdA was treated with GSH and unbound GSH was removed by gel-filtration. Solid line reports the spectrum of 2 µM RhdA alone. (**C**) Tryptophan fluorescence emission spectra (λ_ex_ = 280 nm) of 2 µM RhdA alone in high-saline concentrated buffer (50 mM Tris-HCl, 1 M NaCl) (solid line) and in the presence of 0.25 mM GSH (dotted line). Spectrum of 2 µM RhdA in the presence 0.25 mM thiosulfate is reported as a positive control (dashed line). (**D**) LC/ESI Q-ToF analysis of RhdA/GSH complex prepared by 20 min incubation (25°C) of RhdA with 8-fold molar excess GSH, followed by desalting on C4-chromatographic pipet tip. Molecular mass range of the deconvoluted ESI spectra of RhdA (grey line) and RhdA/GSH complex (black line) is shown.

Taking advantage of the peculiar position of five tryptophan residues around the active site structure of RhdA [12], intrinsic fluorescence changes [27] were monitored in order to investigate the *in vitro* interaction of RhdA with GSH. As shown in **Figure** **4A**, the intrinsic fluorescence of RhdA was quenched in the presence of GSH. The involvement of the active site Cys^230^ thiol of RhdA in the interaction, suggested by the *in silico* analysis, was proved using a site-directed mutant of RhdA in which the active-site cysteine was substituted by alanine (RhdA-Cys^230^Ala). In this case no fluorescence change was observable in the presence of GSH (**Figure** **4B**).

The nature of the RhdA/GSH complex was investigated by fluorescence approaches and MS-analyses. Measurements of thermally-induced changes of intrinsic fluorescence (**Figure** **5A**) showed that transition temperature of the RhdA/GSH complex was higher than that monitored with RhdA alone, corroborating the conformational stability of the RhdA/GSH complex.

Quenching of the intrinsic RhdA fluorescence due to the interaction with GSH was evident also when fluorescence measurement was preceded by a gel-filtration step, and was not affected by the presence of the reducing agents Tris(2-carboxyethyl)phosphine (TCEP) and DTT (**Figure** **5B**). In the presence of high ionic strength, the GSH-dependent changes of the RhdA fluorescence were not detected (**Figure** **5C**), suggesting that formation of the RhdA/GSH complex is driven by electrostatic interactions. As shown in **Figure** **5C**, high ionic strength did not affect the quenching of RhdA intrinsic fluorescence when the ligand was thiosulfate (i.e. the sulfane sulfur donor of RhdA).

Formation of a covalent complex between RhdA and GSH was definitely excluded by MS analyses (**Figure** **5D**). The MS spectra of mixtures of RhdA and GSH did not show significant difference with respect to the spectra of RhdA alone. In both cases a main species at 31054.2 Da was identified, as expected from RhdA sequence (31054.0 Da).

**Figure 6:**
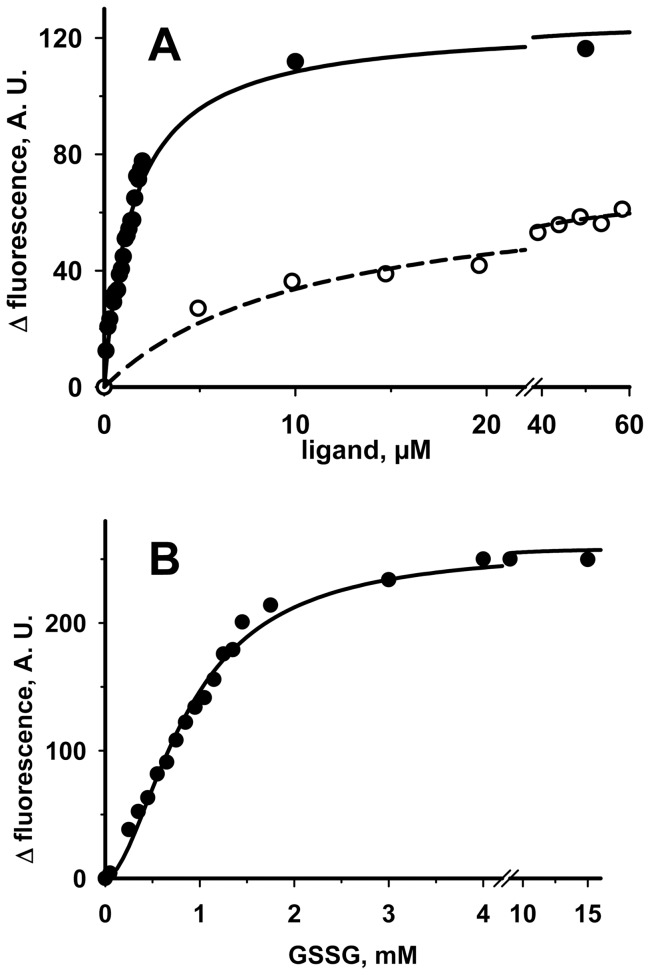
Changes of the intrinsic fluorescence of RhdA upon titration with glutathione species. The amounts indicated in abscissa of GSH (**A**, full circles), GS^•^ (**A**, empty circles), and GSSG (**B**, full circles) were successively added to 2 µM RhdA in 50 mM Tris-HCl, 100 mM NaCl (pH 7.4). Fluorescence intensity was measured at 340 nm (λ_ex_ = 280 nm), and was corrected for dilution.

In order to investigate whether RhdA could interact with other gluthatione species, we used the above fluorimetric approach. The evidence that quenching of RhdA fluorescence was detected also in the presence of either GSSG or glutathione-thiyl radical (GS^•^), an oxidized form of GSH, prompted us to deepen the interaction behaviours of RhdA with these glutathione species. Based on the fluorimetric analyses shown in **Figures** **6A and B**, the estimated *K*_d_ figures of the RhdA/GSH, RhdA/GS^•^ and RhdA/GSSG complexes were 1.5±0.1 µM, 11±2 µM and 1.1±0.1 mM, respectively. The binding of GSSG to RhdA (**Figure** **6B**) appears to be very weak, as compared to that of GSH, this latter appearing to be a “good” ligand for RhdA, also considering that a *K*_d_ of 0.76±0.01 µM was found by fluorimetric analysis of the binding of thiosulfate with RhdA. We are aware that the *K*_d_ figure for GS^•^ could be rougly estimated because of the likely presence of residual GSH in the GS^•^-generation mixture.

**Figure 7:**
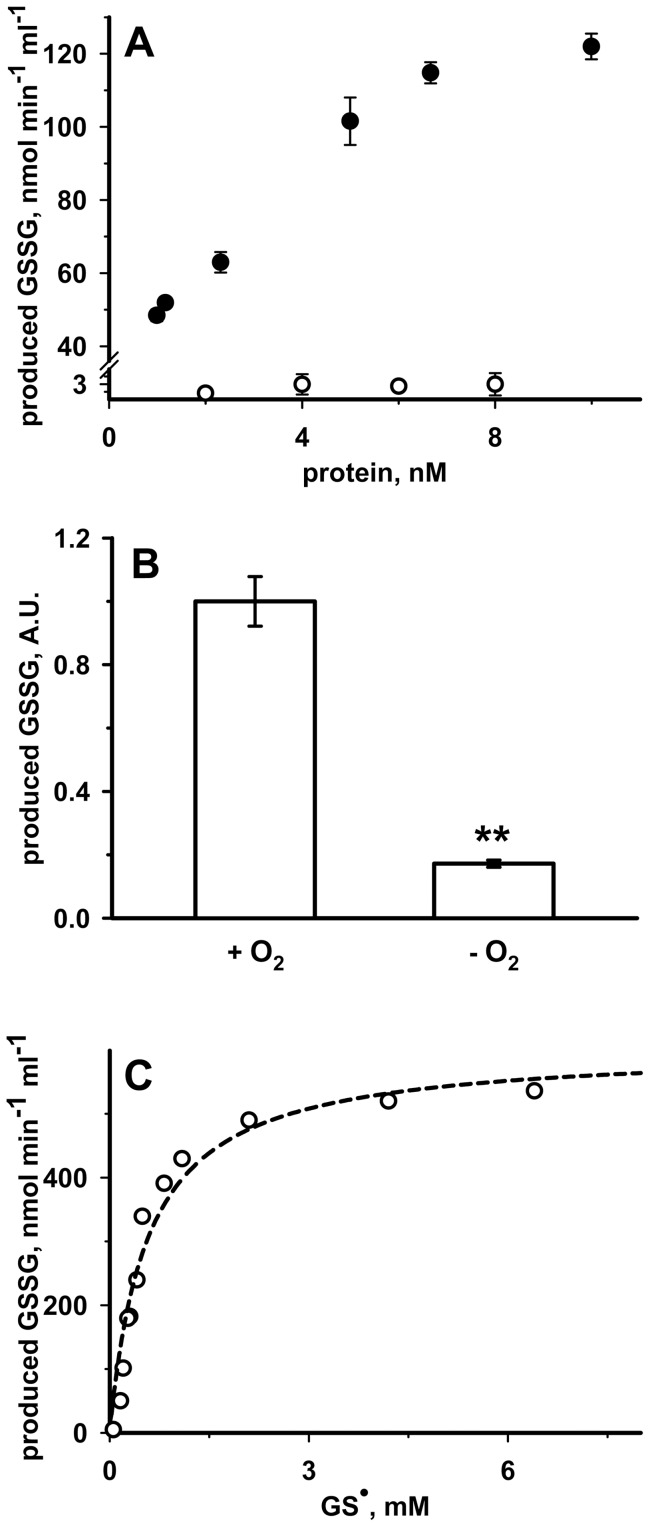
GS^•^-scavenging activity of RhdA. Initial rates of the RhdA-mediated production of glutathione disulphide (GSSG) in the presence of GS^•^ were spectrophotometrically (λ = 340 nm; ε = 6220 M^−1^ cm^−1^) determined by measuring the NADPH consumed in the glutathione oxido-reductase coupled reaction. Reactions were carried out in 50 mM sodium-phosphate buffer (pH 7.4) at 25°C. (**A**) GSSG production in the presence of various concentrations of RhdA (full circles) and RhdA-Cys^230^ (empty circles) is reported. (**B**) GS^•^-scavenging activity of 16 nM RhdA in the absence (−O_2_) of oxygen is reported relatively to that in standard conditions (+O_2_) taken as unitary. A. U., arbitrary units. (**C**) Michaelis-Menten of GS^•^-scavenging activity of RhdA. Best non-linear fit to experimental data is showed (dashed line).

**Figure 8:**
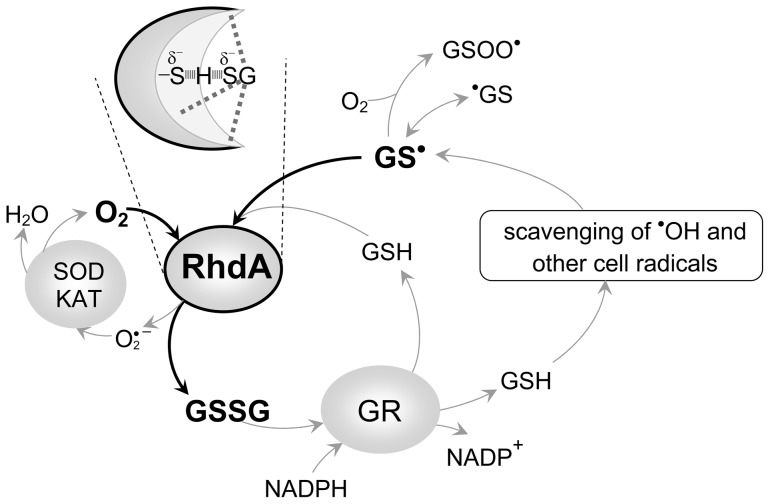
Role of RhdA in GS^•^-scavenging. The scheme displays a possible interplay of RhdA-catalyzed GS^•^-scavenging (thick lines) with other players (grey lines) involved in GSH homeostasis. The oxygen-dependent GS^•^-scavenging activity of RhdA would allow the dissipation of GS^•^ as disulfide glutathione (GSSG), and GSH is produced from GSSG by the glutathione reductase activity (GR). The superoxide anion can be removed by the generally recognized players in superoxide scavenging machinery (i.e. superoxide dismutase, SOD, and catalase, KAT). The inset depicts the likely **feature** of the RhdA active-site at the beginning of the GS^•^-scavenging reaction.

The picture that emerged from these results makes the interactions of RhdA with GSH and GS^•^ a possible key point to provide insights on the mechanism by which RhdA did counteract the oxidative imbalance in *A. vinelandii* shown by the phenotypic characterization of the RhdA null mutant (see above and [14], [15]).

### Glutathione-thiyl radical scavenging activity of RhdA

Considering that thiyl radicals (RS^•^) have been suggested to be important intermediate oxidants during biological conditions of oxidative stress [37], we focussed our study on the interaction of RhdA with the glutathione-thiyl radical (GS^•^). GS^•^ is generated by reaction of GSH with hydroxyl radical (^•^OH), and an “enzymatic” pathway [32] is needed to recover GS^•^ as GSSG, the substrate of glutathione reductase in order to regenerate GSH. In this process, a key step is considered the stabilization of a disulfide anion radical intermediate that facilitates the conversion of GS^•^ to GSSG. Good player in the glutathione-thiyl radical scavenging activity are proteins (e.g. human glutaredoxin, Grx1 [32]) bearing thiols with low p*K*_a_ (∼3.5).

In RhdA, the only cysteine residue, Cys^230^, is mandatory for the correct stabilization of the catalytic pocket structure [12], and its presence in the cysteinate form is favoured by the strong electrostatic field of the catalytic pocket [12]. The electrostatic interactions with active-site residues (Ser^236^, Arg^235^, His^234^, and Thr^232^) influence the p*K*_a_ of the RhdA-Cys^230^ thiol that was predicted to be p*K*_a_ = 3.7 by the algorithm PROPKA.

We, therefore, reasoned that the peculiar property of the active site thiol of RhdA along with the interaction behaviour with glutathione (see above), could make RhdA a catalyst for GS^•^-scavenging activity. To test this hypothesis, GSSG production *via* RhdA in the presence of a GS^•^-generation system was studied. Rate of GSSG production was RhdA concentration dependent (**Figure** **7A**), and it was linear from 1 nM to 6–7 nM RhdA with a turnover of ∼500 s^−1^. At higher RhdA concentration the high reaction speed made it difficult to measure the initial velocity. Control experiments showed that generation of GSSG by RhdA in the presence of GSH alone was negligible, being turnover ∼0.01 s^−1^. The reaction was negligible when the site-directed mutant RhdA-Cys^230^Ala was used (**Figure** **7A**), thus indicating that the RhdA-Cys^230^ residue is involved in the GS^•^-scavenging activity of RhdA. The ability of RhdA to generate GSSG from GS^•^ was significantly diminished in conditions in which molecular oxygen was depleted (**Figure** **7B**) suggesting the participation of molecular oxygen in the RhdA-catalyzed reaction.

To further elucidate the efficiency of RhdA in the GS^•^-scavenging activity, kinetic studies were performed, and, as shown in **Figure** **7C**, the reaction appeared to follow a classical Michaelis-Menten kinetics. Calculations of apparent kinetic parameters gave the following figures: *K*_m_ = 0.56±0.11 mM, *k*_cat_ = 629±44 s^−1^, *k*_cat_/*K*_m_ = 1.12×10^6^ M^−1^ s^−1^.

## Discussion

The phenotypic features of the *A. vinelandii* RhdA-null mutant (MV474) defined in our previous works [14], [15], [36] are consistent with a working model in which defects in antioxidant systems are related to the lack of expression of this rhodanese-like protein. None of the other rhodanese-like proteins present in *A. vinelandii* shows structural features (e.g. sequence catalytic stretch) similar to those of RhdA, and the present work steamed from the characterization of the MV474 strain. In particular, we demonstrated that in the absence of RhdA an increased production of ROS occurred during cellular respiration [15], and that, to rescue the effects of RhdA lack, *A. vinelandii* needed to activate defensive activities against oxidative damage.

In the present study, further insights on the effects of the oxidative imbalance generated by the absence of RhdA (e.g. increased levels of lipid hydroperoxides, formation of DTT-irreversible modified forms of glutathione) were provided indicating that defensive mechanisms activated in MV474 are not adequate to counteract the absence of RhdA. Starting from the evidence that the reshuffling of GSH was impaired in MV474, a possible link between RhdA and homeostasis of reduced glutathione (GSH) was investigated. In particular, the ability of RhdA to counteract DTT-irreversible oxidation of GSH was evidenced in growth conditions where oxidative stress was elicited by the presence of phenazine methosulfate. Therefore, in this study it was investigated whether interactions of RhdA with glutathione species could be a clue feature to explain the altered cellular redox equilibrium of MV474. The central role of GSH in mediating the dissipation of radicals within cells does involve intermediate formation of the glutathione-thiyl radical (GS^•^) [38]–[41], and during conditions of oxidative stress, like in the RhdA-null mutant strain, formation of GS^•^ can be considered an important intermediate oxidant [37]. In order to regenerate GSH, GS^•^ has to be converted to GSSG, the substrate of glutathione reductase [32]. Production of GSSG from GS^•^ after reaction with GSH and oxygen is disfavoured at neutral pH [25], [37], whereas could be favoured in the presence of protein players bearing thiols with low pK_a_. This “enzymatic” reaction was *in vitro* described for the human glutaredoxin (*h*Grx1), for which biological GS^•^-scavenging activity was claimed [32].

The evidence that RhdA is able to bind GSH with high affinity, and that the binding of GSSG to RhdA appears to be very weak, as compared to that of GSH, indicated that RhdA could be an efficient catalyst in the GS^•^-scavenging activity. Moreover, the peculiar structure of its active-site loop around the thiol of Cys^230^, the only cysteine residue in RhdA, seems to be tailored for productive interaction of RhdA with glutathione species that were abolished in the presence of the RhdA-Cys^230^Ala mutant. In the presence of GS^•^-generating system, RhdA catalyzes GSSG formation showing a turnover ∼180-fold higher than that of *h*Grx1, the glutaredoxin with the highest GS^•^-scavenging turnover [32], [42]. Moreover, the catalytic efficiency of RhdA in the conversion of GS^•^ to GSSG is highlighted by considering that nM concentrations of RhdA were used as catalyst, instead of the µM concentrations used in the case of *h*Grx1 [32].

The proposed catalytic mechanism of GS^•^-scavenging by glutaredoxin(s) [32] does imply the stabilization of the radical giving a Grx-SSG^•-^ disulfide anion radical intermediate, followed, in the presence of molecular oxygen, by the formation of Grx-SSG. Although intermediates in the catalytic mechanism of GS^•^-scavenging by RhdA were not detected, the extent of acceleration of GSSG formation by RhdA deserves some considerations. In the present study, the affinity for RhdA of both GSH and GSSG was measured, and we found that the *K*_d_ value for RhdA/GSH complex was ∼700-fold lower than that for RhdA/GSSG complex. Somehow, the binding of GSH to RhdA easily occurs, thus favouring formation of the low-affinity leaving group GSSG. To our knowledge, affinity data for glutathione binding to human glutaredoxins are not available whereas a low-affinity *K*_d_ value (8.6 mM) was estimated for GSH binding to the *Populus tremula* Grx C4 [43]. The measured high affinity of RhdA for GSH, along with the depicted orientation and proximity of GSH thiol and RhdA-Cys^230^ thiol shown in the *in silico* model (**Figure** **3**), might explain the catalytic efficiency of RhdA in GS^•^-scavenging.

Taking into account that in *A. vinelandii* the concentration of glutathione is ∼2 mM (estimated from our data and from [44]), it is likely that *in vivo* RhdA is mostly charged with GSH in the thiolate form (GS^-^) due to the strong electropositive environment of the RhdA active site. It can be envisaged that RhdA improves the reactivity of glutathione with GS^•^ by increasing the concentration of GS^-^ at neutral pH without recurring to formation of the protein-glutathione disulfide anion radical intermediate whose reduction could be a step that limits the overall reaction.

Considering that the fate of GS^•^ in the cells will reflect the kinetics of reactions that produce and remove this radical, the performance of RhdA in GS^•^-scavenging activity should have physiologic impact in *A. vinelandii*. The radical GS^•^ can be converted to GSSG, or can undergo also either overoxidation to glutathione peroxyl radical [24] or intramolecular hydrogen transfer to form carbon-centered radicals [25]. By evaluating control reactions in the assay condition, nanomolar concentration of RhdA accelerates 7-fold the spontaneous rate of conversion of GS^•^ to GSSG that is achieved before the addition of RhdA in the assay. In the cell, concentration of RhdA is in the micromolar level (i.e. ∼3 µM; estimated by semi-quantitative western blot data), suggesting that RhdA can be a good catalyst for the *in vivo* scavenging of GS^•^.

This work further supports the idea originally developed by studies on mammalian 3-mercaptopyruvate sulfurtransferase [45] that some of the ubiquitous rhodanese-like proteins can be versatile players of cellular redox regulation, and highlights an interplay between the ability in managing antioxidant defence and peculiarity of their active-site structure. In particular, as depicted in the scheme presented in **Figure** **8**, the key-point of the RhdA-mediated GS^•^-scavenging activity is the efficient reaction of GS^•^ with the RhdA catalytic pocket, proved by the detected apparent kinetic parameters. The proposed scheme overlays with the general accepted mechanism in which the superoxide anion is considered an “intracellular radical sink” [46], and it does not include the role of ascorbate in radical scavenging [37] on the basis of the followings. Some primates and prokaryotes, with some exceptions, are not known to synthesize ascorbate [47] and, noticeably, genes coding for key enzymes of the ascorbate biosynthesis are lacking in the *A. vinelandii* genome, thus highlighting biological relevance of RhdA as GS^•^-scavenger.

Previous works [13], [28] were focussed on the RhdA property to stabilize a persulfide sulfur on the catalytic cysteine residue (Cys^230^) highlighting a role of RhdA in sulfane sulfur mobilization. It can be supposed that the RhdA-Cys^230^ thiol functionally acts according to its state: persulfurated or not. This aspect deserves further work that should combine persulfide sulfur with radical chemistries.

## Supporting Information

Table S1 **Effect of RhdA lack on levels of cysteine and l-glutamate, and on glutathione disulfide reductase (GR) activity in** ***A. vinelandii*** **.** Significant differences between the strains at the level of *P*<0.05 and *P*<0.01 (Student's *t* test) are indicated as * and **, respectively. All values represent means ± standard deviation (SD) for three independent determinations. ^a^ Taken from Cartini et al. (2011).
(DOC)

Method S1 **Supporting methods and references for Table S1.**
(DOC)

## References

[1] Westley J, Adler H, Westley L, Nishida C (1983) The sulfurtransferases. Fundam Appl Toxicol 3: 377–382. 6357923 10.1016/s0272-0590(83)80008-6

[2] Pagani S, Bonomi F, Cerletti P (1984) Enzymic synthesis of the iron-sulfur cluster of spinach ferredoxin. Eur J Biochem 142: 361–366. 6430704 10.1111/j.1432-1033.1984.tb08295.x

[3] Nandi DL, Westley J (1998) Reduced thioredoxin as a sulfur-acceptor substrate for rhodanese. Int J Biochem Cell Biol 30: 973–977. 9785461 10.1016/s1357-2725(98)00050-8

[4] Nandi DL, Horowitz PM, Westley J (2000) Rhodanese as a thioredoxin oxidase. Int J Biochem Cell Biol 32: 465–473. 10762072 10.1016/s1357-2725(99)00035-7

[5] Mikami Y, Shibuya N, Kimura Y, Nagahara N, Ogasawara Y, et al (2011) Thioredoxin and dihydrolipoic acid are required for 3-mercaptopyruvate sulfurtransferase to produce hydrogen sulfide. Biochem J 439: 479–485. 21732914 10.1042/BJ20110841

[6] Williams RA, Kelly SM, Mottram JC, Coombs GH (2003) 3-Mercaptopyruvate sulfurtransferase of Leishmania contains an unusual C-terminal extension and is involved in thioredoxin and antioxidant metabolism. J Biol Chem 278: 1480–1486. 12419809 10.1074/jbc.M209395200

[7] Ray WK, Zeng G, Potters MB, Mansuri AM, Larson TJ (2000) Characterization of a 12-kilodalton rhodanese encoded by *glpE* of *Escherichia coli* and its interaction with thioredoxin. J Bacteriol 182: 2277–2284. 10735872 10.1128/jb.182.8.2277-2284.2000 PMC111278

[8] Westrop GD, Georg I, Coombs GH (2009) The mercaptopyruvate sulfurtransferase of *Trichomonas vaginalis* links cysteine catabolism to the production of thioredoxin persulfide. J Biol Chem 284: 33485–33494. 19762467 10.1074/jbc.M109.054320 PMC2785193

[9] Papenbrock J, Guretzki S, Henne M (2011) Latest news about the sulfurtransferase protein family of higher plants. Amino Acids 41: 43–57. 20135153 10.1007/s00726-010-0478-6

[10] Bordo D, Bork P (2002) The rhodanese/Cdc25 phosphatase superfamily. Sequence-structure-function relations. EMBO Rep 3: 741–746. 12151332 10.1093/embo-reports/kvf150 PMC1084204

[11] Colnaghi R, Pagani S, Kennedy C, Drummond M (1996) Cloning, sequence analysis and overexpression of the rhodanese gene of *Azotobacter vinelandii* . Eur J Biochem 236: 240–248. 8617271 10.1111/j.1432-1033.1996.00240.x

[12] Bordo D, Deriu D, Colnaghi R, Carpen A, Pagani S, et al (2000) The crystal structure of a sulfurtransferase from *Azotobacter vinelandii* highlights the evolutionary relationship between the rhodanese and phosphatase enzyme families. J Mol Biol 298: 691–704. 10788330 10.1006/jmbi.2000.3651

[13] Cartini F, Remelli W, Dos Santos PC, Papenbrock J, Pagani S, et al (2011) Mobilization of sulfane sulfur from cysteine desulfurases to the *Azotobacter vinelandii* sulfurtransferase RhdA. Amino Acids 41: 141–150. 20213443 10.1007/s00726-010-0529-z

[14] Cereda A, Carpen A, Picariello G, Tedeschi G, Pagani S (2009) The lack of rhodanese RhdA affects the sensitivity of *Azotobacter vinelandii* to oxidative events. Biochem J 418: 135–143. 18925874 10.1042/BJ20081218

[15] Remelli W, Cereda A, Papenbrock J, Forlani F, Pagani S (2010) The rhodanese RhdA helps *Azotobacter vinelandii* in maintaining cellular redox balance. Biol Chem 391: 777–784. 20482308 10.1515/BC.2010.073

[16] Bordo D, Forlani F, Spallarossa A, Colnaghi R, Carpen A, et al (2001) A persulfurated cysteine promotes active site reactivity in *Azotobacter vinelandii* rhodanese. Biol Chem 382: 1245–1252. 11592406 10.1515/BC.2001.155

[17] Giles GI, Jacob C (2002) Reactive sulfur species: an emerging concept in oxidative stress. Biol Chem 383: 375–388. 12033429 10.1515/BC.2002.042

[18] Kiley PJ, Storz G (2004) Exploiting thiol modifications. PLoS Biol 2: e400. 15547642 10.1371/journal.pbio.0020400 PMC526781

[19] Poole LB, Karplus PA, Claiborne A (2004) Protein sulfenic acids in redox signaling. Annu Rev Pharmacol Toxicol 44: 325–347. 14744249 10.1146/annurev.pharmtox.44.101802.121735

[20] Krivobok S, Kuony S, Meyer C, Louwagie M, Willison JC, et al (2003) Identification of pyrene-induced proteins in *Mycobacterium* sp. strain 6PY1: evidence for two ring-hydroxylating dioxygenases. J Bacteriol 185: 3828–3841. 12813077 10.1128/JB.185.13.3828-3841.2003 PMC161579

[21] Venkatraman A, Landar A, Davis AJ, Chamlee L, Sanderson T, et al (2004) Modification of the mitochondrial proteome in response to the stress of ethanol-dependent hepatotoxicity. J Biol Chem 279: 22092–22101. 15033988 10.1074/jbc.M402245200

[22] Santos PM, Benndorf D, Sa-Correia I (2004) Insights into *Pseudomonas putida* KT2440 response to phenol-induced stress by quantitative proteomics. Proteomics 4: 2640–2652. 15352239 10.1002/pmic.200300793

[23] Florczyk MA, McCue LA, Stack RF, Hauer CR, McDonough KA (2001) Identification and characterization of mycobacterial proteins differentially expressed under standing and shaking culture conditions, including Rv2623 from a novel class of putative ATP-binding proteins. Infect Immun 69: 5777–5785. 11500455 10.1128/IAI.69.9.5777-5785.2001 PMC98695

[24] Sevilla MD, Becker D, Yan M (1990) The formation and structure of the sulfoxyl radicals RSO^•^, RSOO^•^, RSO_2_ ^•^, and RSO_2_OO^•^ from the reaction of cysteine, glutathione and penicillamine thiyl radicals with molecular oxygen. Int J Radiat Biol 57: 65–81. 1967295 10.1080/09553009014550351

[25] Zhao R, Lind J, Merenyi G, E. Eriksen T (1997) Significance of the intramolecular transformation of glutathione thiyl radicals to α-aminoalkyl radicals. Thermochemical and biological implications. J Chem Soc, Perkin Trans 2: 569–574.

[26] Newton JW, Wilson PW, Burris RH (1953) Direct demonstration of ammonia as an intermediate in nitrogen fixation by Azotobacter. J Biol Chem 204: 445–451. 13084615

[27] Pagani S, Forlani F, Carpen A, Bordo D, Colnaghi R (2000) Mutagenic analysis of Thr-232 in rhodanese from *Azotobacter vinelandii* highlighted the differences of this prokaryotic enzyme from the known sulfurtransferases. FEBS Lett 472: 307–311. 10788632 10.1016/s0014-5793(00)01477-0

[28] Forlani F, Cereda A, Freuer A, Nimtz M, Leimkuhler S, et al (2005) The cysteine-desulfurase IscS promotes the production of the rhodanese RhdA in the persulfurated form. FEBS Lett 579: 6786–6790. 16310786 10.1016/j.febslet.2005.11.013

[29] Sipe HJ, Corbett JT, Mason RP (1997) In vitro free radical metabolism of phenolphthalein by peroxidases. Drug Metab Dispos 25: 468–480. 9107547

[30] Griffiths G, Leverentz M, Silkowski H, Gill N, Sanchez-Serrano JJ (2000) Lipid hydroperoxide levels in plant tissues. J Exp Bot 51: 1363–1370. 10944149

[31] Riemenschneider A, Nikiforova V, Hoefgen R, De Kok LJ, Papenbrock J (2005) Impact of elevated H_2_S on metabolite levels, activity of enzymes and expression of genes involved in cysteine metabolism. Plant Physiol Biochem 43: 473–483. 15914014 10.1016/j.plaphy.2005.04.001

[32] Starke DW, Chock PB, Mieyal JJ (2003) Glutathione-thiyl radical scavenging and transferase properties of human glutaredoxin (thioltransferase). Potential role in redox signal transduction. J Biol Chem 278: 14607–14613. 12556467 10.1074/jbc.M210434200

[33] Bradford MM (1976) A rapid and sensitive method for the quantitation of microgram quantities of protein utilizing the principle of protein-dye binding. Anal Biochem 72: 248–254. 942051 10.1016/0003-2697(76)90527-3

[34] Bas DC, Rogers DM, Jensen JH (2008) Very fast prediction and rationalization of pK_a_ values for protein-ligand complexes. Proteins 73: 765–783. 18498103 10.1002/prot.22102

[35] Yoon SJ, Park JE, Yang JH, Park JW (2002) OxyR regulon controls lipid peroxidation-mediated oxidative stress in *Escherichia coli* . J Biochem Mol Biol 35: 297–301. 12297013 10.5483/bmbrep.2002.35.3.297

[36] Cereda A, Carpen A, Picariello G, Iriti M, Faoro F, et al (2007) Effects of the deficiency of the rhodanese-like protein RhdA in *Azotobacter vinelandii* . FEBS Lett 581: 1625–1630. 17383639 10.1016/j.febslet.2007.03.028

[37] Wardman P (1995) Reactions of thiyl radicals. In: Cadenas E, editor. Biothiols in Health and Disease. New York: Marcel Dekker Inc. 1–19.

[38] Quintiliani M, Badiello R, Tamba M, Esfandi A, Gorin G (1977) Radiolysis of glutathione in oxygen-containing solutions of pH7. Int J Radiat Biol Relat Stud Phys Chem Med 32: 195–202. 302250 10.1080/09553007714550891

[39] Schreiber J, Foureman GL, Hughes MF, Mason RP, Eling TE (1989) Detection of glutathione thiyl free radical catalyzed by prostaglandin H synthase present in keratinocytes. Study of co-oxidation in a cellular system. J Biol Chem 264: 7936–7943. 2470737

[40] Bradshaw TP, McMillan DC, Crouch RK, Jollow DJ (1995) Identification of free radicals produced in rat erythrocytes exposed to hemolytic concentrations of phenylhydroxylamine. Free Radic Biol Med 18: 279–285. 7744312 10.1016/0891-5849(94)e0136-7

[41] Kwak HS, Yim HS, Chock PB, Yim MB (1995) Endogenous intracellular glutathionyl radicals are generated in neuroblastoma cells under hydrogen peroxide oxidative stress. Proc Natl Acad Sci U S A 92: 4582–4586. 7753847 10.1073/pnas.92.10.4582 PMC41988

[42] Gallogly MM, Starke DW, Leonberg AK, Ospina SM, Mieyal JJ (2008) Kinetic and mechanistic characterization and versatile catalytic properties of mammalian glutaredoxin 2: implications for intracellular roles. Biochemistry 47: 11144–11157. 18816065 10.1021/bi800966v PMC3569056

[43] Noguera V, Walker O, Rouhier N, Jacquot JP, Krimm I, et al (2005) NMR reveals a novel glutaredoxin-glutaredoxin interaction interface. J Mol Biol 353: 629–641. 16181638 10.1016/j.jmb.2005.08.035

[44] Fahey RC, Brown WC, Adams WB, Worsham MB (1978) Occurrence of glutathione in bacteria. J Bacteriol 133: 1126–1129. 417060 10.1128/jb.133.3.1126-1129.1978 PMC222142

[45] Nagahara N (2011) Intermolecular disulfide bond to modulate protein function as a redox-sensing switch. Amino Acids 41: 59–72. 20177947 10.1007/s00726-010-0508-4

[46] Winterbourn CC (1993) Superoxide as an intracellular radical sink. Free Radic Biol Med 14: 85–90. 8384151 10.1016/0891-5849(93)90512-s

[47] Arrigoni O, De Tullio MC (2002) Ascorbic acid: much more than just an antioxidant. Biochim Biophys Acta 1569: 1–9. 11853951 10.1016/s0304-4165(01)00235-5

