# Supplementary material for: Involvement of the Azotobacter vinelandii Rhodanese-Like Protein RhdA in the Glutathione Regeneration Pathway
Source: PLoS One. 2012 Sep 25;7(9):e45193. doi: 10.1371/journal.pone.0045193 (PMC3458005; doi:10.1371/journal.pone.0045193)
Supplement: Table S1 — Effect of RhdA lack on levels of cysteine and l-glutamate, and on glutathione disulfide reductase (GR) activity in A. vinelandii . Significant differences between the strains at the level of P<0.05 and P<0.01 (Student's t test) are indicated as * and **, respectively. All values represent means ± standard deviation (SD) for three independent determinations. a Taken from Cartini et al. (2011). (DOC) [file pone.0045193.s001.doc]

**Table S1. Effect of RhdA lack on levels of cysteine and l-glutamate, and on glutathione disulfide reductase (GR) activity in *A. vinelandii*.**

Significant differences between the strains at the level of *P* < 0.05 and *P* < 0.01 (Student’s *t* test) are indicated as * and **, respectively. All values represent means ± standard deviation (SD) for three independent determinations. ^a^ Taken from Cartini et al. (2011).

| Carbon source | strain | cysteine  (pmol mg^-1^ cells) |  | l-glutamate  (nmol mg^-1^ cells) |  | GR activity  (U mg^-1^ protein) |  |
| --- | --- | --- | --- | --- | --- | --- | --- |
| Sucrose | UW136 | 10.3 ± 1.4^a^ |  | 77 ± 9 |  | 109 ± 10 |  |
|  | MV474 | 22.0 ± 1.8^a^* |  | 67 ± 5 |  | 91 ± 12 |  |
| Gluconate | UW136 | 9.5 ± 2.4 |  | 142 ± 19 |  | 77 ± 4 |  |
|  | MV474 | 70.9 ± 13.4** |  | 197 ± 27* |  | 65 ± 10 |  |
