# Supplementary material for: Involvement of the Azotobacter vinelandii Rhodanese-Like Protein RhdA in the Glutathione Regeneration Pathway
Source: PLoS One. 2012 Sep 25;7(9):e45193. doi: 10.1371/journal.pone.0045193 (PMC3458005; doi:10.1371/journal.pone.0045193)
Supplement: Method S1 — Supporting methods and references for Table S1. (DOC) [file pone.0045193.s002.doc]

**Supplementary Methods.**

Measurement of the levels of cysteine were performed by a monobromobimane HPLC method (Riemenschneider et al., 2005) using 50 mg (fresh weight) cell samples. This method measures cumulatively both the dithiothreitol- (DTT-) reducible and the reduced forms of free cysteine.

L-Glutamate was detected by an enzymatic method based on the use of glutamate dehydrogenase. Cell-free extracts were prepared from 300-mg (fresh weight) cell aliquots by sonication (five 30 s pulses with intermitted one-min-cooling periods in Soniprep 150, UK) in 50 mM Tris-HCl, 100 mM NaCl (pH 7.4; 900 µl), and cell debris was removed by centrifugation (30 min at 10000 g). Reaction was initiated adding 2.8 U of bovine liver glutamate dehydrogenase (type II, 40 U mg-1; Sigma) to mixture containing sample (50 µl; ~750 µg protein), 1 mM NAD^+^ in 50 mM Tris-HCl, 100 mM NaCl (pH 7.4; final volume 1 ml). Absorbance at 340 nm (ε = 6220 M^-1^ cm^‑1^) was recorded for 3 min and subtracted from the absorbance of a control reaction carried out omitting NAD^+^. In order to validate the method, control reactions were performed replacing the sample with L-glutamic acid (20 nM).

Glutathione disulfide reductase activity assay, based on GSH reduction of 5,5’-dithiobis-(2-nitrobenzoic acid) (DTNB), was carried out according to Smith et al. (1988) with minor modifications. Assay mixture (1 ml) containing 0.14 mM NADPH, 0.43 mM GSSG, 0.45 mM DTNB, and cell-free sample (20-30 µl; ~80 µg protein) in 50 mM potassium phosphate, 100 mM NaCl (pH 7.4) was incubated at 25 °C. The reaction was started with GSSG and the increase at 412 nm was monitored (ε = 13600 M^-1^ cm^-1^). Rate of control reactions carried out in the absence of GSSG was subtracted. One unit (U) of GSSG reductase activity is defined as the enzyme amount that consumes 1 µmol GSSG per min at 25 °C.

**Supplementary References.**

Cartini F, Remelli W, Dos Santos PC, Papenbrock J, Pagani S, et al. (2011) Mobilization of sulfane sulfur from cysteine desulfurases to the *Azotobacter vinelandii* sulfurtransferase RhdA. Amino Acids 41: 141-150

Riemenschneider A, Nikiforova V, Hoefgen R, De Kok LJ, Papenbrock J (2005) Impact of elevated H_2_S on metabolite levels, activity of enzymes and expression of genes involved in cysteine metabolism. Plant Physiol Biochem 43: 473-483

Smith IK, Vierheller TL, Thorne CA (1988) Assay of glutathione reductase in crude tissue homogenates using 5,5'-dithiobis(2-nitrobenzoic acid). Anal Biochem 175: 408-413
